# Rotational symmetry engineered, polarization and incident angle-insensitive, perfect metamaterial absorber for X and Ku band wireless applications

**DOI:** 10.1038/s41598-022-07824-x

**Published:** 2022-03-08

**Authors:** Saif Hannan, Mohammad Tariqul Islam, Sami H. A. Almalki, Mohammad Rashed Iqbal Faruque, M. Salaheldeen M, Md. Shabiul Islam

**Affiliations:** 1 Department of Electrical, Electronic and Systems Engineering, Universiti Kebangsaan Malaysia (UKM), 43600 Bangi, Selangor Malaysia; 2 Electrical Engineering Department, College of Engineering, University of Ha’il, Ha’il, 81481 Saudi Arabia; 3 Department of Electrical Engineering, College of Engineering, Taif University, P.O. Box 11099, Taif, 21944 Kingdom of Saudi Arabia; 4 Space Science Center (ANGKASA), University Kebangsaan Malaysia (UKM), 43600 Bangi, Selangor Malaysia; 5 Department of Electrical Engineering, Faculty of Energy Engineering, Aswan University, Aswan, 81528 Egypt; 6 Faculty of Engineering, Multimedia University (MMU), 63100 Cyberjaya, Selangor, Malaysia

**Keywords:** Engineering, Materials science

## Abstract

In this paper, a square enclosed split-maze shaped metamaterial absorber is proposed for X and Ku band wireless applications. Two square metal enclosures were introduced around the split-maze structure to make it rotational symmetric and thus insensitive to cross-polarization. The proposed absorber has shown maximum absorptions at 9.33 GHz, 12.83 GHz, 13.86 GHz, and 15.61 GHz with single negative value of permittivity. The absorber is insensitive to the incident angle of applied EM waves for normal and oblique incidence up to 180 degrees. In addition, it was proved co- & cross-polarization insensitive due to the symmetric structure of the patch. A comprehensive equivalent circuit analysis was done to explain the fundamental EM behaviour of the metamaterial structure, and the circuit outputs coincided with the simulation results. Finally, the metamaterial was measured for both unit cell, and the array after fabrication and simulation results were validated. The proposed MMA is suitable for wireless applications in devices, especially for sensing, EM energy harvesting, EM coupling reduction, and antenna gain enhancement purposes.

## Introduction

Electromagnetic (EM) metamaterial absorbers (MMA) have become an interesting field of research among scientists and engineers in the last two decades. The versatility of the usage of EM metamaterials in the field of communication devices, sensors, health-care devices, energy harvesting tools, electric-automobile sectors, and so on has made metamaterials or MMAs an essential engineering concept. Different types of substrate materials along with various patch resonators and solid ground on opposite faces of the substrates are proposed as EM absorbers, with or without metamaterial properties. Generally, an EM absorber is claimed as an MMA if it represents a negative value of either permittivity or permeability at the applied EM waves through it^1^. Usually, split-ring or complementary split-rings are used as a patch for these MMA^2^. Hundreds of designs have been proposed to date using split-ring or complementary split-ring resonators as MMA. The split-ring resonator is the most common approach to attain MM characteristics and absorptions^3^. Thus, improvisation in patch designs of MMA using split-rings or modified split-rings is done at recent times with optimum performance indicators.

The MMAs designed with modified split-ring based patch has shown better performances like maximum absorptions at resonance frequencies with MM characteristics, the greater value of the effective medium ratio (EMR)^4^, incident angle-insensitivity^5^, ultra-broadband absorption, etc. Although these MMAs have diversity in patch designs, only a few are perfect MMA. A perfect MMA should be insensitive to the incident angle of the applied EM wave and the co- & cross-polarization of EM wave^6,7^. Incident angle-insensitiveness can be achieved by oppositely oriented split-ring or modified split-ring resonators on the patch design, but insensitivity to both co- & cross-polarization^8^ elements of the applied EM waves is rare to find. Thus, almost all these proposed absorbers are co-polarized MMA or cross-polarization converter^9^. Co-polarized MMA are capable of absorption of the applied EM waves by half or less than half of the energy while the rest of the energy cannot be absorbed by them due to cross-polarization elements of the incident EM wave. Thus, they are not perfect absorber and considered as cross-polarization converter.

Perfect MMA is needed to absorb EM waves (at resonance frequencies) effectively so that they can be proposed for different practical applications like EM energy harvesting^10^, EM coupling reduction in communication devices^11^, radar cross-section reduction^12^, isolation of unwanted interference among target frequencies or devices^13,14^, reduction of EM wave-induced heat^15^ and so on.

Perfect MMA can drastically enhance the performance of antennas, sensors, couplers, and satellites. As the world is moving towards 5G or 6G communication systems, the necessity of perfect MMA is inevitable. 5G or 6G devices will be operated at high or very high frequencies which will need control over EM waves for different purposes that can be served by these MMA^16,17^. Thus, the designing and proposition of perfect MMA are recent trends in microwave or millimeter-wave EM applications. Since the perfect MMA can absorb the entire EM wave, the patch structure should be either 90 degrees rotational symmetric or 90 degrees split-spot symmetric^7,18,19^. Such structured perfect MMA are rare to find. This paper proposes a square enclosed split-maze shaped (SE-SMS) metamaterial absorber for X and Ku band applications. The proposed metamaterial is incident angle- and polarization-insensitive and has shown four distinct resonance frequencies with maximum absorptions and metamaterial characteristics.

### The proposed metamaterial structure and its performance

**Figure 1:**
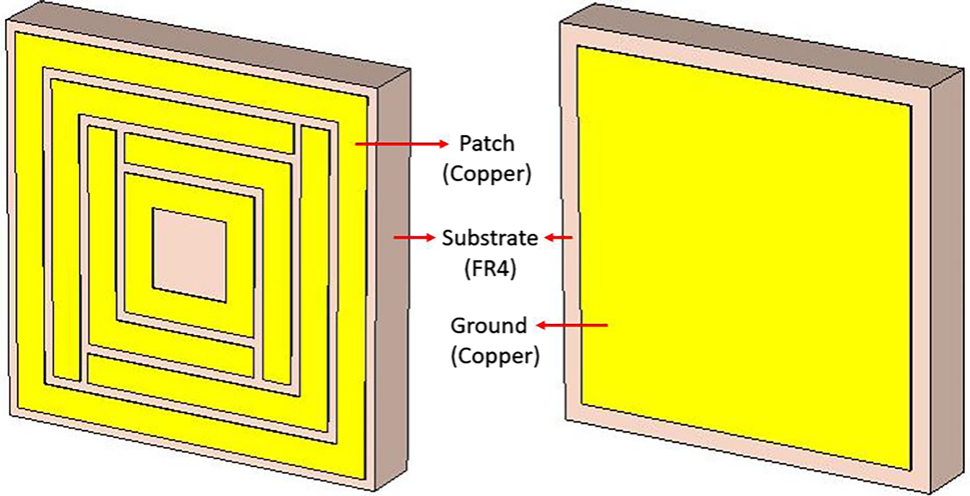
The proposed square enclosed split-maze shaped (SE-SMS) metamaterial absorber.

**Figure 2:**
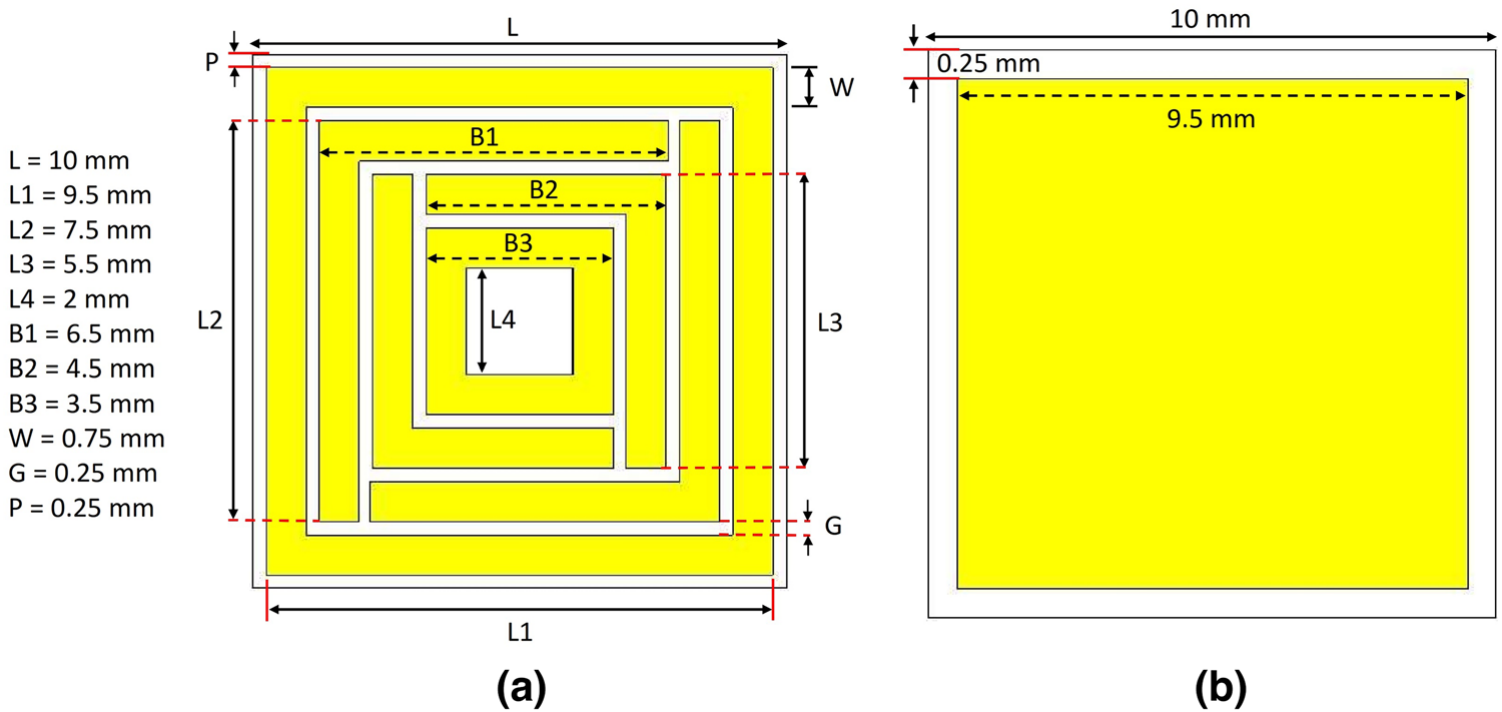
The detailed dimension of (**a**) the patch and (**b**) the ground of the unit cell.

The structure of the proposed square enclosed split-maze shaped (SE-SMS) metamaterial absorber unit cell is shown in Fig. 1. Two square metal enclosures were introduced around the split-maze structure to make it rotational symmetric and thus insensitive to cross-polarization. This unique characteristic of the patch design made it a perfect MMA, which is not present in any literature. It is based on commercially available FR4 substrate (1.6 mm thick) and annealed copper on both sides. Two square perimeters enclose two mutually orthogonal split-maze structures at the outer and inner circumferences, and the ground is cut at the rim. The detailed dimension of the unit cell is shown in Fig. 2.

**Figure 3:**
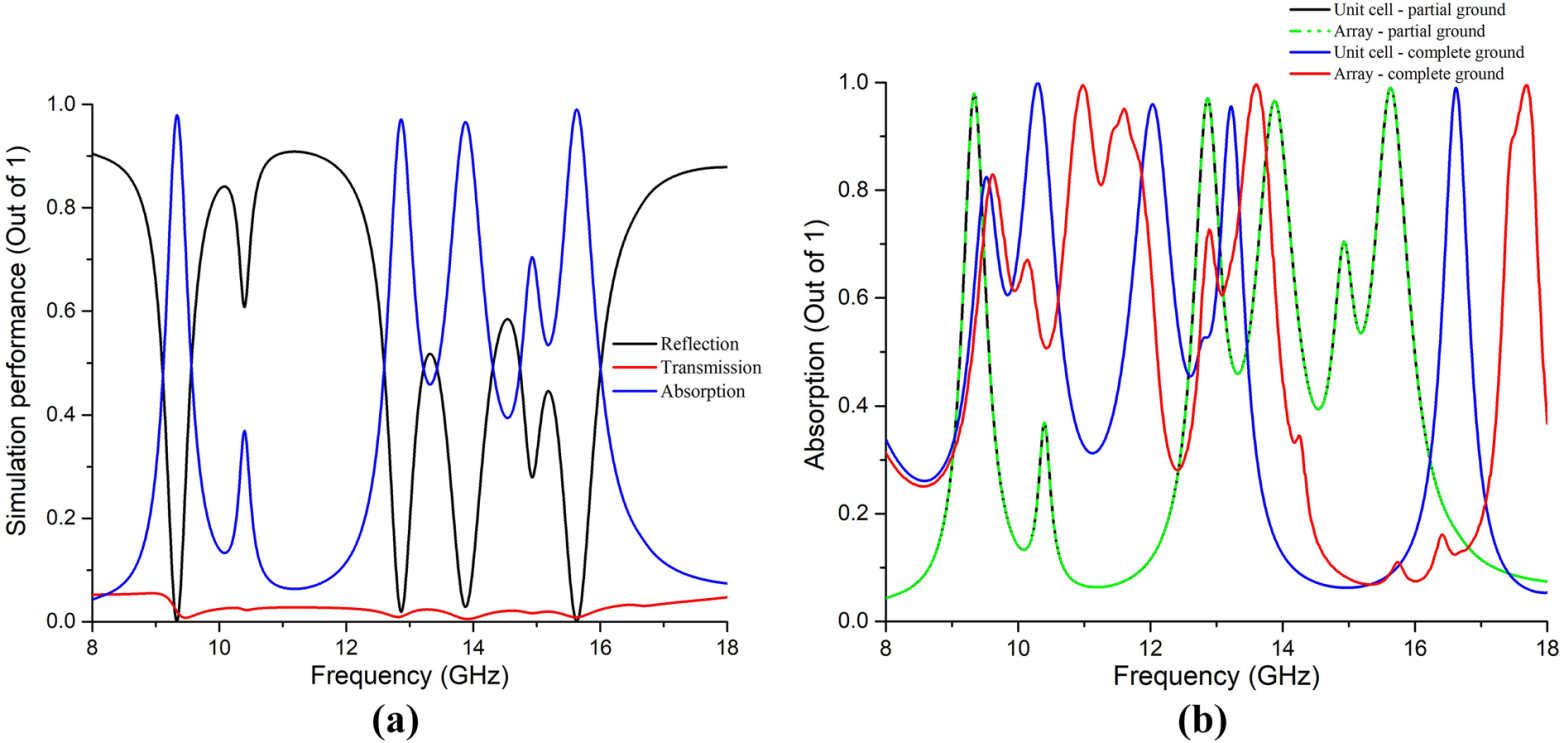
(**a**) Absorption performance of the unit cell with the proposed defected ground, (**b**) comparison of absorption for full ground and defected ground for both the unit cell and the array absorber.

It can be seen from Fig. 2 that the patch has two rotational symmetric square-shaped perimeters at the circumference and the center. Two mutually opposite split-maze structures represent the active resonators between the perimeters. The circumferential square perimeter is 0.25 smaller than the substrate of the unit cell, and the ground is also 0.25 mm less at the circumferential area from the substrate. It was done to avoid mutual coupling among the unit cells in a complete array of absorber^20^. If the ground is equal to the substrate area and the circumferential area of the patch is equivalent to substrate area, it will show absorption and other MM characteristics for a unit cell, but for some unit cells (as matrix) as an array, it will deviate from the properties displayed in case of the unit cell due to sharing of inductance, capacitance and surface current distribution among adjacent unit cells (Fig. 3b). Two neighboring unit cells must be separated by an adequate inductance and capacitance so that the array shows similar performance as the unit cell (Fig. 3b).

The absorption performance of the unit cell is shown in Fig. 3a. The resonances are found at 9.33 GHz, 12.83 GHz, 13.86 GHz, and 15.61 GHz. At these frequencies, the values of the reflection and the transmission coefficients are found near zero. Primarily, absorption was calculated by the usual formula as in Eq. (1).

Absorption = 1—Reflection cefficient – transmission coefficient

Or

1 $${\text{A}} = {1}{-\!\!-}\left| {S_{11} } \right|^{2} - |S_{21} |^{2}$$

Usually, two waveguide ports are used on both surfaces of the MMA to determine the S parameters. The scattering (S) parameters (S_11_ and S_21_) can be understood as follows:

2 $${\text{S}}_{11} = { }\frac{{\sqrt {{\text{Power reflected from port }}1} }}{{\sqrt {{\text{Power incident on port }}1} }}$$

3 $${\text{S}}_{21} = { }\frac{{\sqrt {{\text{Power delivered to port }}2} }}{{\sqrt {{\text{Power incident on port }}1} }}$$

The S parameters can be defined in terms of electric field^21^. The computed electric field E_c_ on the port consists of the excitation plus the reflected field. The S parameters are understood by

4 $$S_{11} = \frac{{\mathop \smallint \nolimits_{Port 1} \left( {\left( {E_{c} - E_{1} } \right).E_{1}^{*} } \right)dA_{1} }}{{\mathop \smallint \nolimits_{Port 1} \left( {E_{1} .E_{1}^{*} } \right)dA_{1} }}$$

5 $$S_{21} = \frac{{\mathop \smallint \nolimits_{Port 2} \left( {E_{c} .E_{2}^{*} } \right)dA_{2} }}{{\mathop \smallint \nolimits_{Port 2} \left( {E_{2} .E_{2}^{*} } \right)dA_{2} }}$$

where E_1_ and E_2_ are the electric field patterns on port 1 and port 2.

**Figure 4:**
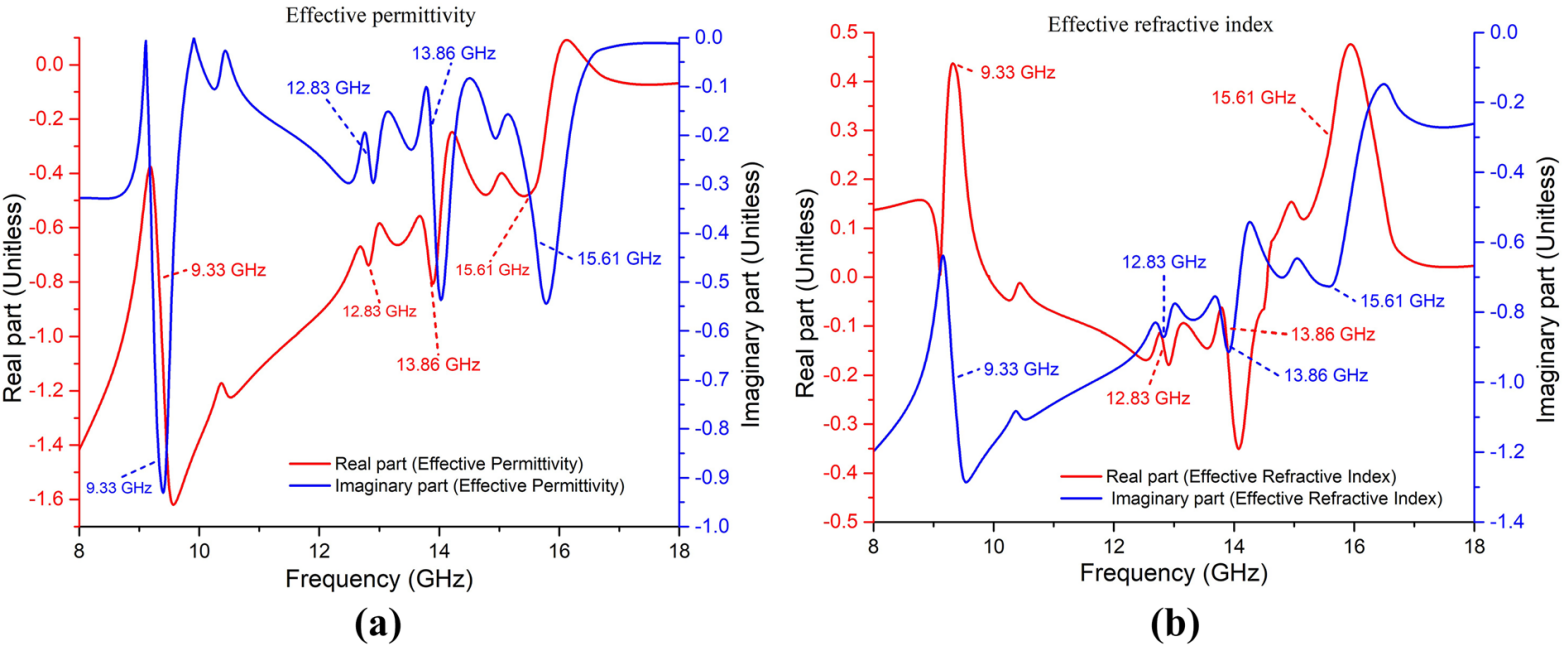
The effective value of (**a**) permittivity and (**b**) refractive index at the operating frequencies of the unit cell.

The permittivity values are found negative (single negative – SNG), and two out of four refractive index values are found negative at the resonance frequencies, as can be understood from Fig. 4. The values are calculated from the absolute value of complex numbers considering real and imaginary parts.

The relative permittivity (or permittivity) is defined as

6 $$\in_{r} = \frac{2}{{\sqrt { - { }\frac{\omega }{c}d} }}{ }\frac{{1 - (S_{21} + S_{11} )}}{{1 + (S_{21} + S_{11} )}}$$

where ${\upomega }$ is the frequency of the applied EM wave, d = thickness of the substrate, and c = speed of light.

The refractive index ŋ is defined as

7 $$\upeta = {\text{ real }}\left[ {\frac{{\text{c}}}{{{\text{i }}\uppi {\text{f d }}}}{ }\sqrt {\frac{{\left( {{\text{S}}_{21} - 1} \right)^{2} - \left( {{\text{S}}_{11} } \right)^{2} }}{{\left( {{\text{S}}_{21} - 1} \right)^{2} + \left( {{\text{S}}_{11} } \right)^{2} }}} } \right]$$

**Figure 5:**
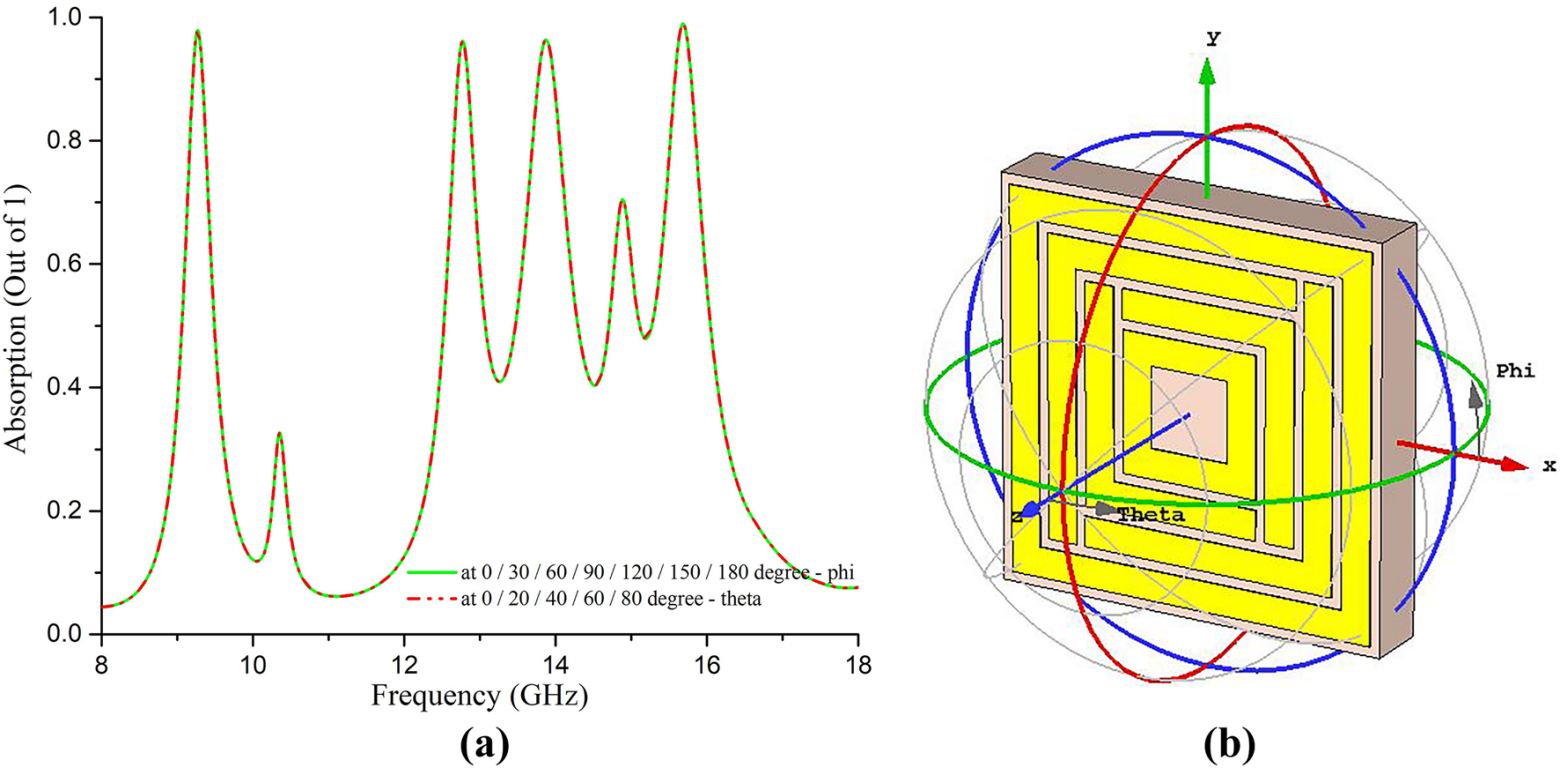
(**a**) Absorption performance of the unit cell at all possible angles for normal and oblique incidence of EM waves, (**b**) graphical representation of theta and phi polarization angles over the unit cell.

The resonance frequencies from the simulation were found at 9.33 GHz, 12.83 GHz, 13.86 GHz, and 15.61 GHz. The negative values of permittivity (at all resonance frequencies) and refractive index (at 12.83 GHz and 13.86 GHz) shown in Fig. 4 ensure the metamaterial characteristics of the proposed SE-SMS absorber. The unit cell was simulated with plane polarization of EM wave at both normal (phi-polarization) and oblique (theta-polarization) incidences, and the absorption was found similar for those polarizations. The absorption performance at all possible normal and oblique incidence is shown in Fig. 5a. Figure 5b is the reference for showing theta and phi polarization of the incident plane waves on the unit cell, considering the plane EM wave propagates along the z-direction through the unit cell. As the unit cell is rotational symmetric by 180 degrees along phi for normal incidence, an elevation of 30 degrees was considered for phi polarization up to 180 degrees. Similarly, for oblique incidence, the top face (patch) of the unit cell can be exposed to incident EM waves up to 90 degrees and thus considered theta polarization with elevation by 30 degrees up to 90 degrees (Fig. 5b). It is evident from Fig. 5a that the unit cell of the proposed structure is incident-angle insensitive for absorption performance.

**Figure 6:**
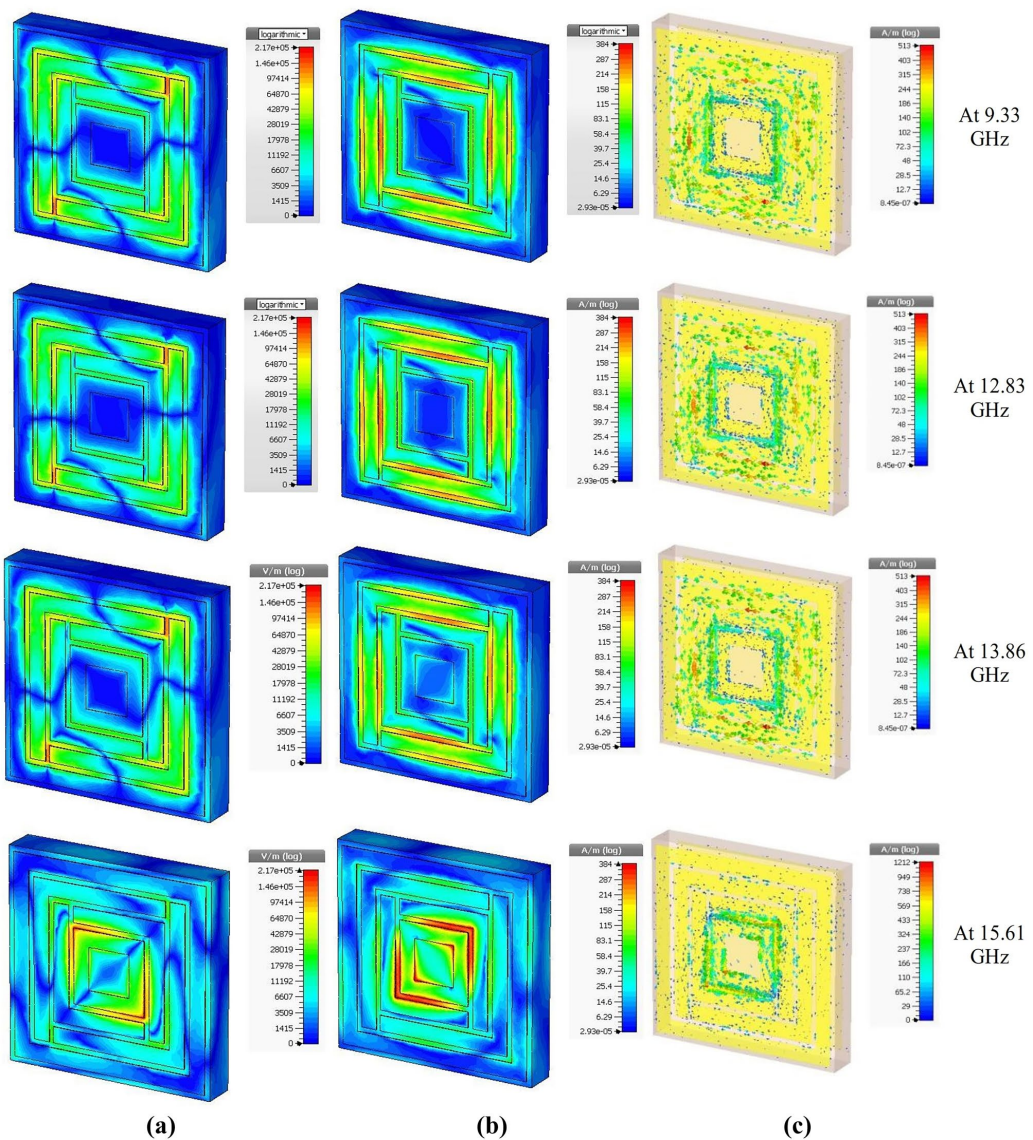
The distribution of (**a**) electric field, (**b**) magnetic field, and (**c**) surface current at the resonance frequencies on the unit cell.

The excitation of the unit cell at resonance frequencies can be better understood by the electric field, magnetic field, and surface current distributions, depicted in Fig. 6. Since the direction of the magnetic field is at right angles to the electric field, the excitation due to the magnetic field can be found at right angles to that of the electric field. The relation among the electric field, magnetic field, and surface current is found from second Maxwell’s equation, as

8 $$\nabla \times H = J + \in \frac{\partial E}{{\partial t}}$$

Which states that the change of electric field at a frequency (with respect to time) times the permittivity plus the current density at that resonance frequency is equal to the change of magnetic field at a perpendicular or right angle to the electric field. In this case, it obeys the second Maxwell’s equation. The surface current distribution shows the agitated portions of the patch resonator at the resonance frequencies, the same as those of electric field distributions. The relation between electric field and surface current is defined as

9 $$J = \sigma E$$

**Figure 7:**
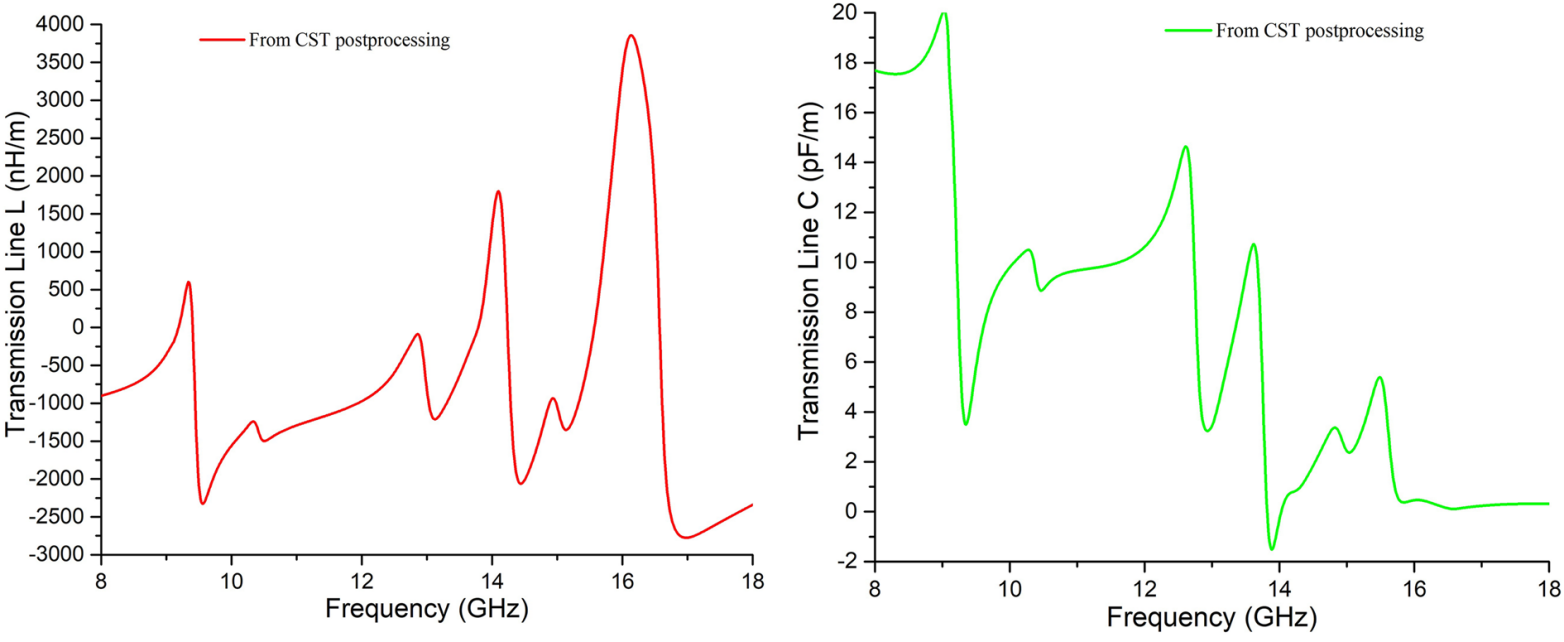
L and C found from transmission line analysis by template-based post-processing of the unit cell.

Thus, the changes in surface currents are found precisely at the same spots of the unit cell for those of electric fields in Fig. 6. The agitation at lower frequencies (higher wavelength) is found at the circumferential area of the patch, and the central portion was agitated at the higher resonance frequency (lower wavelength), as expected. The surface current distribution helps understand the active inductive part at the resonance frequency. The copper cut between the adjacent copper strips represents the capacitive portion of that resonance frequency. An idea of the amount of L and C can be found from the template-based post-processing of the unit cell on the CST 2017 simulator, shown in Fig. 7. Since there is a relation between inductance and time-dependent current density, thus the agitated portions create inductance (Fig. 6c). The non-agitated sectoral parts create capacitance, which leads to the resonance at the frequencies found from simulation, shown in Fig. 6.

#### The equivalent circuit design of the absorber

**Figure 8:**
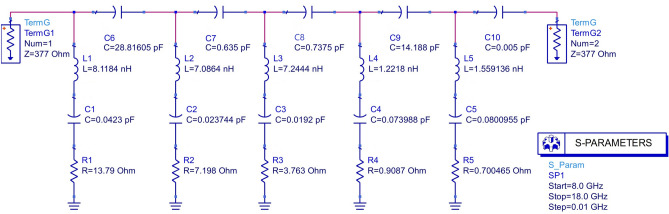
The equivalent circuit of the unit cell.

**Figure 9:**
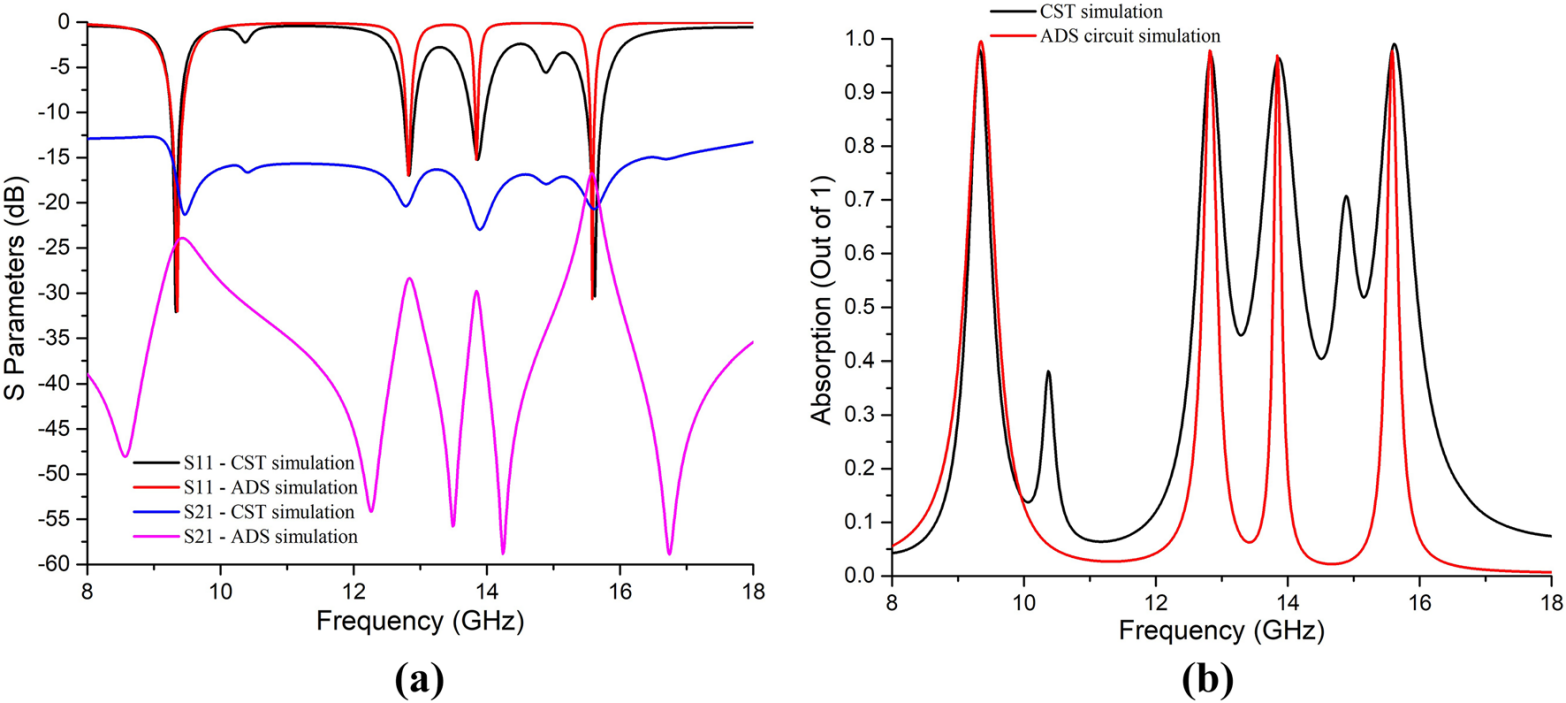
Comparison of (**a**) S-parameters and (**b**) absorption between the ADS equivalent circuit and CST simulation.

The equivalent circuit of the proposed MM unit cell is drawn in Fig. 8. The values of L and C are calculated from Fig. 7 at the resonance frequencies considering the length of L and C from the surface current distribution shown in Fig. 6. The values of R for each L-C-R circuit (for each resonance frequency) were adjusted on the ADS circuit simulator to match the corresponding S_11_ and S_21_ parameters found from the CST simulation. Of the six resonance peaks from CST simulation, only four were chosen for the circuit design. These four resonance frequencies were found with at least -10 dB (equal to 0.1 power gain) values or less, which is the minimum standard S parameter value for MM absorption. Thus, S parameters greater than -10 dB were avoided in the circuit design, as absorption is the issue. This ended with Fig. 9a, b, where the 2nd and the 5th resonance peaks were ignored for the equivalent circuit as per CST simulation. The isolation between two adjacent resonance frequencies was done by placing parallel capacitance (C6, C7, C8, C9 & C10) as shown in Fig. 8. Four parallel L-C-R circuits were required for four resonance frequencies, and the fifth L-C-R circuit was added to satisfy the effect of the ground with the patch through the substrate. The output found from the equivalent circuit matches the CST simulations (Fig. 9). It can be seen from Fig. 9a that the S_11_ parameters from the ADS circuit and CST simulation match reasonably at the resonance frequencies, which shows good agreement for the reflection coefficient. But S_21_ parameters are different except at the lowest and the highest resonance frequencies. This problem is negligible, as the highest (negative) value of S_21_ parameters shows less transmission co-efficient, which is expected for the MMA. Due to the difference in the S_21_ parameter (by ADS circuit), the absorptions are found sharp at the resonance frequencies as per Eq. (1), and thus the absorption bandwidth at the higher frequencies is found less than that of the CST model (Fig. 9b).

**Figure 10:**
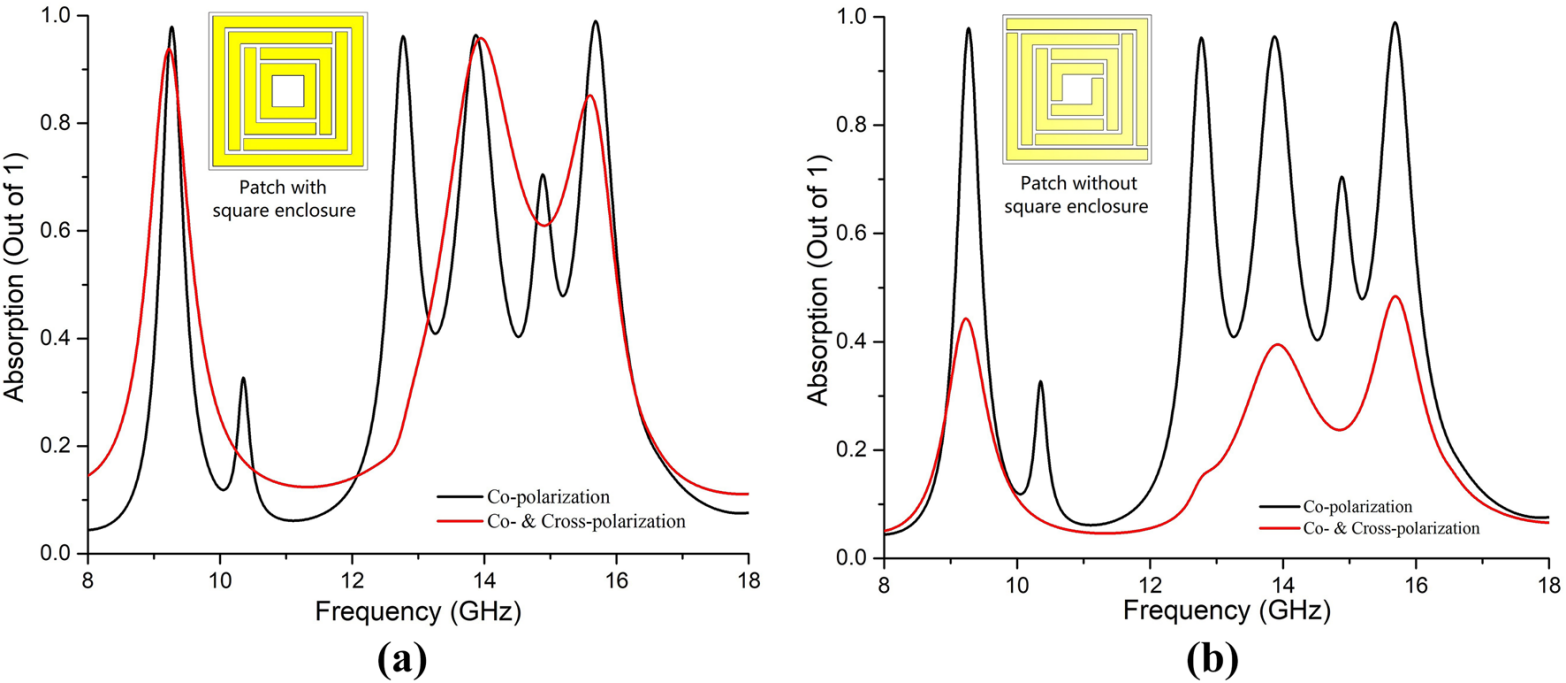
Absorption performance considering both co- and cross-polar EM waves for (**a**) the proposed square enclosed split-maze shaped (SE-SMS) patch structure, and (**b**) without square enclosed split-maze shaped patch structure.

The unit cell has shown insensitivity to both co- & cross-polarization elements of the applied EM waves at three resonance frequencies at X- and Ku- bands, depicted in Fig. 10. Floquet ports were considered in simulation to get co- & cross-polar S parameters. The absorption performance was calculated considering both co- & cross-polar elements of the S parameters as Eq. 10^22^, below.

10 $$A = 1 - |S_{11}^{Co} |^{2} - |S_{11}^{Cross} |^{2} - |S_{21}^{Co} |^{2} - |S_{11}^{Cross} |^{2}$$

It is evident from Fig. 10a that the proposed SE-SMS metamaterial absorber acts as a perfect metamaterial absorber at three distinct frequencies (9.33 GHz, 13.86 GHz, and 15.6 GHz) in X and Ku bands, with the proposed SE-SMS patch structure of the metamaterial absorber. The perfect absorption (in terms of absorption of both co-polar & cross-polar incident EM waves) was achieved due to the symmetrical patch structure achieved by the proposed unique square enclosures. In addition, these enclosures helped only to get absorption for cross-polarized waves. They didn’t influence the resonating sector of the patch surface at resonance frequencies which can be understood from surface current distributions shown in Fig. 6.

Moreover, the structure has shown the same response without the square enclosures considering only the co-polarized EM waves but didn’t show expected absorption for both co-polar & cross-polar calculation as per Eq. (10), as shown in Fig. 10b. Thus, the square enclosures are deemed necessary for the perfect MM absorption. The co-polarization analysis for the SE-SMS patch structure helped understand the MM behavior and necessary analysis.

#### Measurement of the absorber

**Figure 11:**
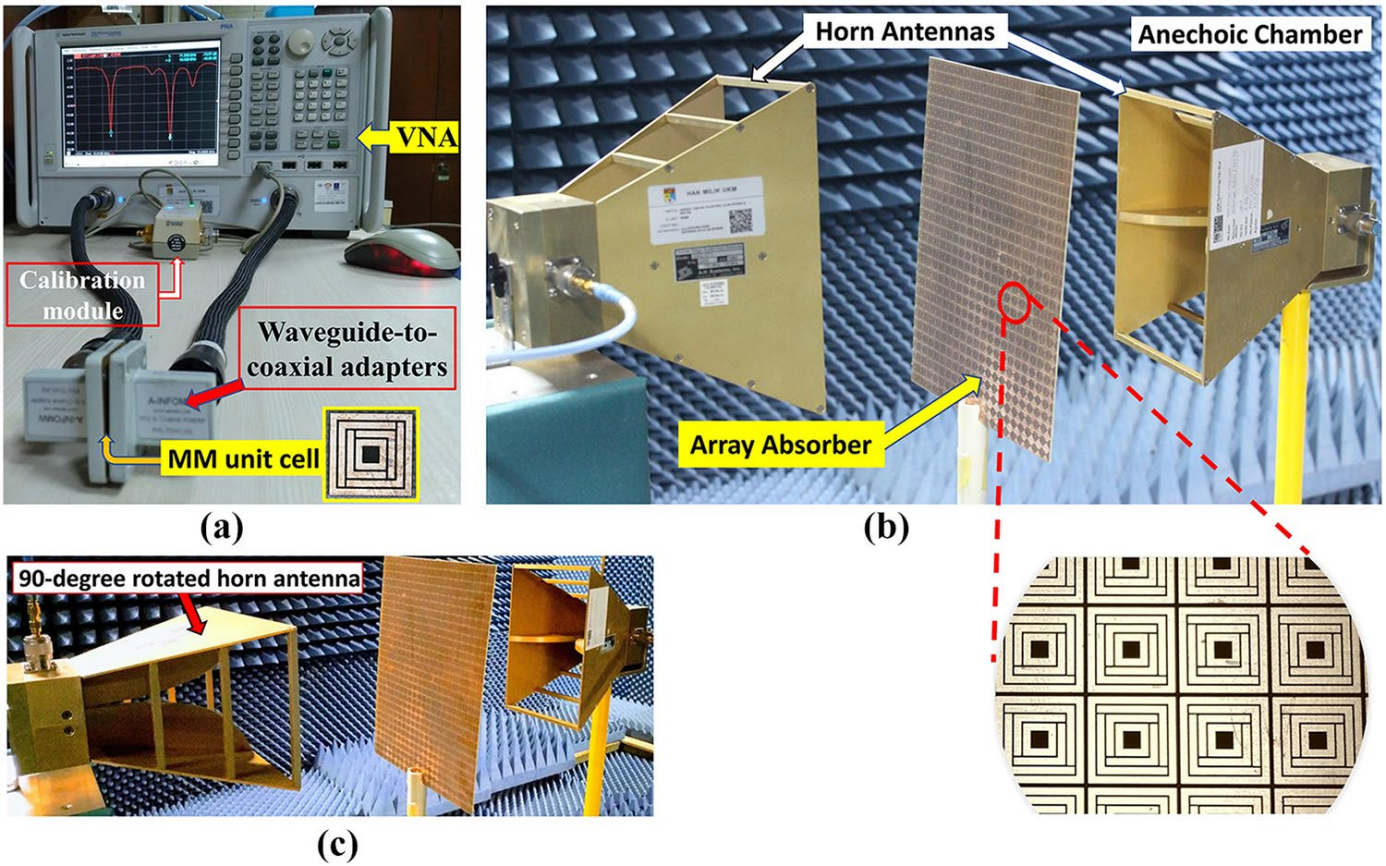
Measurement setup for the proposed SE-SMS absorber as (**a**) unit cell, (**b**) array (co-polarization), and (**c**) array (cross-polarization).

Measurement of MMA (both unit cell and array) is essential to validate the simulation results. The proposed SE-SMS metamaterial was fabricated on an FR4 substrate (thickness 1.6 mm, dielectric constant 4.3). The unit cell (Fig. 11a) was placed in between two waveguide-to-coaxial adapters (or waveguide ports) connected with a vector network analyzer (VNA) [model: Agilent N5227A] to measure S_11_ and S_21_ parameters. The readings were copied for both real and imaginary parts and then computed to compare with corresponding simulation results. The array (an FR4 sheet of A4 size) consisting of a matrix of 21 × 29-unit cells was placed in an anechoic chamber^23^ in between two horn antennas (Fig. 11b) connected with the VNA (outside the chamber) and S_11_ and S_21_ parameters were measured accordingly. Before starting measurement of both unit cell and the array, the setup was calibrated^24^ with Agilent N4694-6001 electronic calibration module (shown in Fig. 11a) for appropriate measurement.

**Figure 12:**
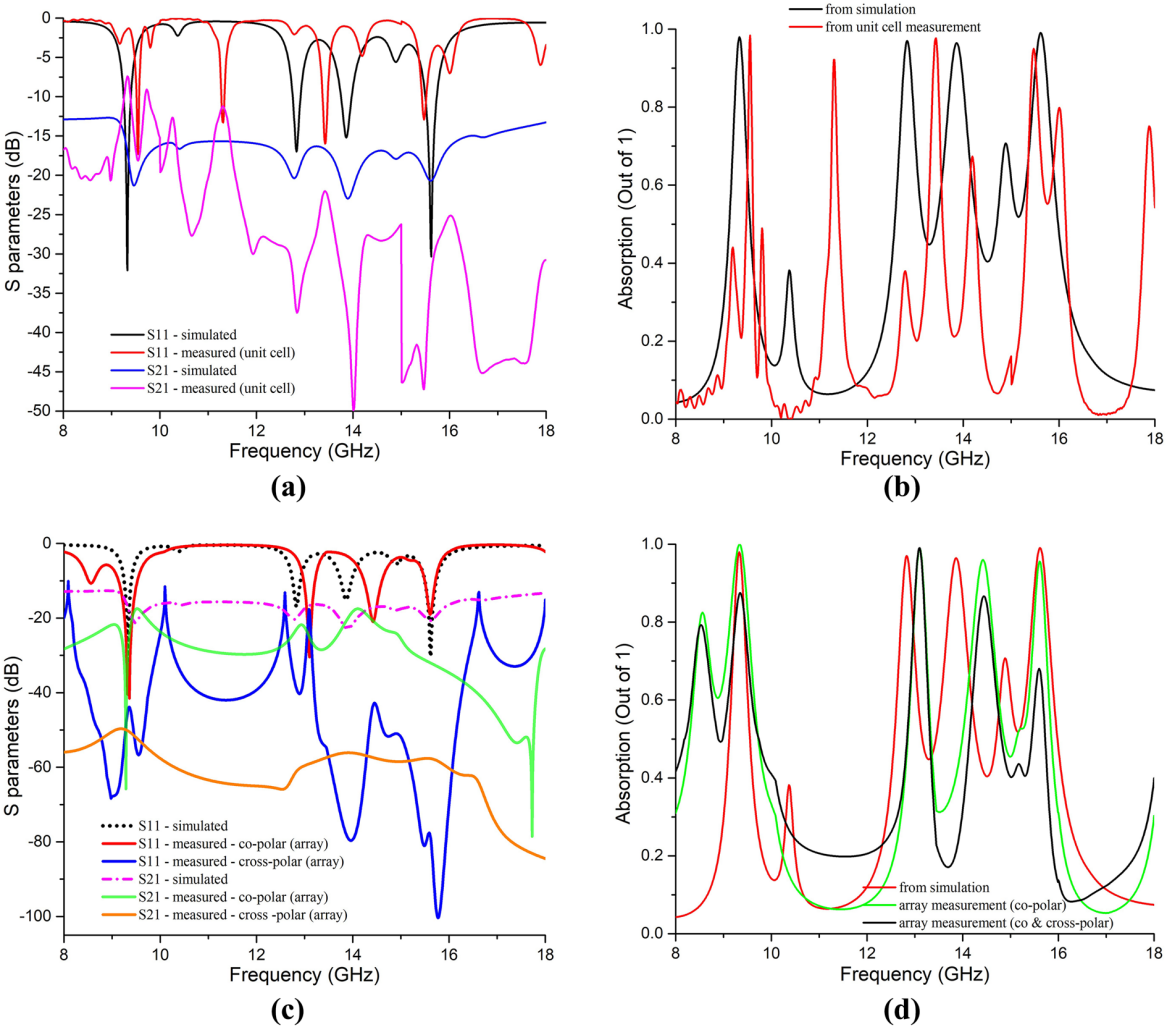
Comparison of simulated and measured values of the unit cell for co-polar (**a**) S-parameters and, (**b**) absorption. Comparison of simulated and co—& cross-polar measured values of the array for (**c**) S-parameters and (**d**) absorption.

The measured S parameters and absorption have mostly coincided with simulation results, depicted in Fig. 12. It is observed that S_11_ parameters are coincided at the lower and upper frequencies but deviate at the region in between the lower and the upper resonance (for the unit cell). It happened due to a practical reason; three different waveguide-to-coaxial adapters^25^ were used to measure the absorber (unit cell) from 8 to 18 GHz, and the data points were added as per simulation data to get the final results, as waveguide ports were available for 7-to-10 GHz, 10-to-15 GHz, and 15-to-22 GHz respectively. During simulation, there were no conjoint (and different) waveguide ports; thus, the data has shown resonance at appropriate frequencies. On the other hand, three different waveguide ports were used during unit cell measurement due to the lack of a single waveguide port set for 8 to 18 GHz. Thus, some non-specific coupling missing among those three waveguide ports caused the resonance frequencies at the middle region to shift from those of simulation (Fig. 12a). Moreover, some negative absorptions were found near 10.5 GHz frequency from the unit cell measurement (Fig. 12b). It happened due to data joining from the 10-to-15 GHz adapter set with the 7-to-10 GHz adapter set. Tracing the ending S parameter values from the 7-to-10 GHz adapter set to the starting S parameter values from the 10-to-15 GHz adapter set didn’t match. Thus, the absorption calculated from S parameters resulted in negative values absorptions near the 10.5 GHz region (Fig. 12b). Negative absorption is due to the data error for setting up different waveguide adapters for the measurement. But it can be neglected as the absorption peak is not expected at this frequency, and it didn’t influence the absorption performance of the proposed MM unit cell at the desired resonance frequencies. In the case of array measurement, only two horn antennas were used for the entire operating frequency (8 GHz to 18 GHz), and thus no unexpected absorptions were found (Fig. 12d). The array was measured inside the anechoic chamber with two horn antennas, as shown in Fig. 11b. The setup shown in Fig. 11b is considered as the measurement of S -parameters for co-polarized EM waves incident on the absorber. As the horn antennas can emit both co- and cross-polar EM waves, the absorber should absorb both co- and cross-polarized incident EM waves to prove it as a perfect absorber as per Fig. 10a. Thus, a cross-polarization setup was also done by rotating the 1st horn antenna at right angles so that the absorber can be considered for cross-polar measurement as depicted in Fig. 11c. The measured values of S-parameters for co-polarized and perfect (considering both co-& cross-polarized) EM waves and the corresponding absorptions are plotted in Fig. 12c, d.

The S_21_ parameters are found at a satisfactory level (less than -10 dB in the operating frequencies), which shows a very low transmission coefficient through the proposed absorber, which is expected due to the solid ground. Thus, the absorption performance by the unit cell (Fig. 12b) is found at four resonance frequencies with a shift in the middle region due to the measured S_11_ parameter shown in Fig. 12a. The measured absorption performance by the absorber considering perfect absorption of both co- and cross-polarized incident EM waves (plotted in Fig. 12d) has shown four resonance peaks, as the corresponding S parameters are found less than -10 dB in Fig. 12c. Thus, the proposed absorber is a perfect metamaterial absorber proved by both simulation and measurement, as per Eq.( 10) for perfect absorption.

## Discussion

The proposed SE-SME metamaterial absorber measurement can be considered to declare it as a perfect MMA. This absorber can be used for perfect absorption, energy harvesting, or sensing applications at X and Ku bands. Performance comparison of the proposed absorber with recent relevant published works may justify its superiority for the usability and appropriateness as a perfect absorber for practical applications, shown in Table 1 below.

It is evident from Table 1 that the proposed absorber is a perfect absorber considering insensitivity to the maximum angle of incidence and cross-polarization. A unique rotational symmetry technique was applied for patch designing to make the absorber a perfect absorber. In addition, it can absorb more than 99% of the applied EM wave effectively. This unique property and single negative (SNG) permittivity value have ensured the absorber is a perfect metamaterial absorber. This metamaterial absorber can be employed in various wireless application-based devices operating at X and Ku band frequencies for many applications like sensing, EM energy harvesting, EM coupling reduction, antenna gain enhancement, etc.

## Conclusion

In this paper, a square enclosed split-maze shaped (SE-SMS) metamaterial absorber is proposed for X and Ku band wireless applications. A unique rotational symmetry technique was applied with two square metal enclosures introduced around the split-maze structure, which eventually made it insensitive to cross-polarization and thus a perfect absorber. Four perfect absorption peaks were achieved at 9.33 GHz, 12.83 GHz, 13.86 GHz, and 15.61 GHz. The proposed metamaterial absorber (MMA) was proved as a perfect MMA considering incident angle-insensitivity for both normal incidence (up to 180 degrees) and oblique incidence (up to 90 degrees). Moreover, it is insensitive to both co-polarized and cross-polarized EM waves. In addition, it shows single negative (SNG) characteristics for permittivity and thus negative value of the refractive index, which ensures the metamaterial characteristics at the resonance frequencies. The equivalent circuit analysis was done to explain the elementary electromagnetic behaviour of the proposed absorber. Finally, the measurement of the absorber unit cell and the array has validated the simulation results. The proposed MMA can be applied to devices of wireless applications at X and Ku bands, especially for sensing, EM energy harvesting, EM coupling reduction, and antenna gain enhancement purposes.

## Methods

### Co-polarization simulation

The unit cell was designed as per Fig. 1 first and then set for simulation on CST 2017. Perfect electric field (E_t_ = 0) was set along the x-axis, and perfect magnetic field (H_t_ = 0) was set along the y-axis. The EM wave propagation was set along the z-axis as open space. Two plane waveguide ports were set at a normal position from the unit cell surfaces along the z-axis as the EM wave sources. Frequency-domain solver with the phase reflection diagram setup was chosen for the simulation.

### Cross-polarization simulation

Two square metal enclosures were introduced inside and outside the split-maze structure to make the unit cross-polarization insensitive, as shown in Fig. 1. Unit cell boundary conditions were set along the x- and y-axis and open space were set along the z-axis during simulation. Floquet ports were set with Z_Max_ and Z_Min_ eigenvalues to apply the EM waves. Cross-polar data were achieved by taking S parameters with notation by SZ_max_Z_Min_ or SZ_Min_Z_Max_ modes. These S parameters were considered as scattering parameters due to cross-polarized EM waves.

### Equivalent circuit setup

An LRC circuit was chosen for each resonance frequency, and a parallel capacitor was chosen between two adjacent LRC circuits to implement the capacitive isolation between two sharp neighboring absorption peaks. Since there is an air gap between the waveguide port to the absorber surface, the impedance of the ports was set at 377 ohms (Fig. 8). To compare the data found from the equivalent circuit with that of CST simulation, the step was set to 0.01 GHz in between the starting (8 GHz) and stop frequency (18 GHz) so that the number of points (total 1001 points) matches with that from the CST simulation. Unlike CST simulation output, only four resonance peaks were targeted during the circuit design, as only -10 dB values were found for four resonances.

**Table 1:** Comparison of performance of the proposed SE-SMS metamaterial absorber.

References	Year	Substrate material	Structural symmetry	Resonance frequencies (GHz)	Unit cell size	Equivalent circuit analysis	Max. absorption (%)	Cross-polarization Insensitivity	Max. co-polarization insensitivity (°)
^26^	2017	FR4	No	8.48, 15.4, 17.81	9 × 9 mm^2^	Not shown	> 95	No	60
^27^	2017	FR4	No	9.46, 13.90	5.5 × 5.5 mm^2^	Not shown	> 99	No	45
^28^	2018	FR4	No	13.78, 15.3	19 × 15 mm^2^	Shown	> 99	No	40
^29^	2019	FR4	No	11.56, 12.27, 14.19	15 × 12 mm^2^	Shown	< 99	No	80
^30^	2019	FR4	No	8.6, 12.8, 17.3	9.2 × 9.2 mm^2^	Not shown	99.9	No	90
^7^	2019	FR4	Yes	11.31, 14.13, 14.23, 14.69, 17.79, 17.81	10 × 10 mm^2^	Shown	98.15	Yes	90
^31^	2020	FR4	Yes	8.23, 10.33, 14.32, 15.58	9.6 × 9.6 mm^2^	Not shown	99.9	No	90
^32^	2020	Polyimide film	Yes	8.5, 13.5, 17	8 × 8 mm^2^	Not shown	99	No	90
^18^	2020	FR4	Yes	11.23, 14.18, 17.37, 19.18	9 × 9 mm^2^	Shown	99.13	Yes	90
^33^	2021	FR4	Yes	8.9, 17.1	9.7 × 9.7 mm^2^	Not shown	> 80	No	90
^34^	2021	FR4	Yes	8.96, 9.93, 10.85, 11.9, 12.9, 13.97, 15, 16.21, 17.95, 19.1	7.2 × 7.2 mm^2^	Shown	96.3	No	60
This work	2021	FR4	Yes	9.33, 12.83, 13.86, 15.61	10 × 10 mm^2^	Shown	> 99	Yes	180
